# Placental Development and Physiological Changes in Pregnant Ewes in Silvopastoral and Open Pasture Systems during the Summer

**DOI:** 10.3390/ani13030478

**Published:** 2023-01-30

**Authors:** Julia Morgana Vieira Dada, Matheus Luquirini Penteado dos Santos, Ana Paula Schneiders Dani, Cecília Paulina Johann Dammann, Letícia Pinto, Frederico Márcio Corrêa Vieira, Flávia Regina Oliveira de Barros

**Affiliations:** 1 Biometeorology Study Group (GEBIOMET), Federal University of Technology—Paraná (UTFPR), Dois Vizinhos 85660-000, Brazil; 2 Coordination of the Bioprocess and Biotechnology Engineering, Federal University of Technology—Paraná (UTFPR), Dois Vizinhos 85660-000, Brazil

**Keywords:** heat stress, sheep production system, sheep pregnancy, placental development

## Abstract

**Simple Summary:**

Heat stress is a physiological condition where an animal fails to adequately dissipate body heat; this results in increased blood flow in the animal’s core and negatively affects its physiological system. Considering this problem, this study aimed to analyze the reproductive and physiological changes in ewes subjected to heat stress during pregnancy. Twenty-four pregnant crossbred ewes were kept at UTFPR-DV’s (Brazil) silvopastoral (SP) or open pasture (OP) systems throughout their pregnancy. During the experiment, microclimatic variables, sheep’s blood samples, and physiological variables were collected every two weeks. After the birth of the lambs, the placentas were also collected. Our results showed that both systems were stressful for the sheep, but the SP system had lower air and grass temperatures than the OP system. The respiratory and heart rates of animals from the OP system were higher than those from the SP system. As regards the animals’ immune cells, their mobilization was not affected by the systems, while the neutrophil count was only affected by time. Regarding placental biometry, it was observed that placentas in twin pregnancies had a greater membrane area. We concluded that the type of production system used affects the thermal comfort of pregnant ewes; an SP system can offer more amenable microclimatic conditions, which result in greater comfort for the ewes.

**Abstract:**

This study aimed to analyze the reproductive and physiological changes in ewes subjected to heat stress during pregnancy at UTFPR-Brazil. Twenty-four pregnant crossbred ewes were kept in a silvopastoral system (SP) or an open pasture system (OP) throughout the final trimester of pregnancy. Both systems were stressful, but the SP system had lower air temperature than the OP system (26.0 ± 0.38 and 26.9 ± 0.41 °C, respectively; *p* = 0.0288). Moreover, the radiant thermal load of the two groups presented a difference of 34 Wm^−2^ (*p* = 0.0288), and the grass temperature was also lower in the SP system compared to that in the OP system (23.4 ± 0.37 and 25.6 ± 0.44 °C, respectively; *p* = 0.0043). The respiratory and heart rates of animals from the OP group were higher than those from the SP group (*p* < 0.001), but no difference was observed in the mobilization of white blood cells (*p* = 0.4777), and the neutrophil count was only affected by time (*p* < 0.0001). As regards placental biometry, placentas in twin pregnancies had a greater membrane area (*p* = 0.0223), but no differences between the systems were observed in placental weight (*p* = 0.1522) and the number of cotyledons (*p* = 0.5457). We concluded that the type of rearing system used affects the thermal comfort of pregnant ewes, and that an SP system can offer more amenable microclimatic conditions, which result in greater comfort for the ewes.

## 1. Introduction

An animal’s environment is defined as comfortable when the animal is in its thermoneutral zone; that is, the heat produced by metabolism (thermogenesis) is lost (thermolysis) to the environment without prejudice to the animal’s performance [1]. When this process does not occur properly, and a higher body temperature is maintained, thermal stress due to heat is created, and it is, therefore, necessary for the animal to use devices capable of restoring its thermal balance with the environment. For sheep, the thermoneutral zone, which is its ideal ambient temperature, is established between 15 and 20 °C [2].

Heat stress is caused by a combination of high temperature and relative humidity. This causes animal behavioural and physiological changes, affecting food and water intake, growth, reproduction, milk production, haematological changes, and concentrations of cortisol and thyroid hormones in plasma [3,4,5].

In warm-blooded animals, behavioural change is the first mechanism for achieving body heat loss when the ambient temperature is high, followed by increased respiratory and heart rates [6]. As noted above, a high ambient temperature results in behavioural and other physiological changes in animals, affecting food and water intake, growth, reproduction, and milk production, in addition to haematological changes and changes in plasma concentrations of cortisol and thyroid hormones [5,7,8].

During pregnancy, the placenta nourishes the developing fetus and transports oxygen to meet its demands. In sheep, the larger the maternal—fetal surface, the greater the placental transport capacity; this is associated with the thickness of the placental barrier and can allow greater exchange of metabolic substrates [9]. As the placenta is the organ through which respiratory gases, nutrients, and wastes are exchanged between the maternal and fetal systems, placental vascular development is thought to play a vital role in ensuring the adequate exchange of nutrients and oxygen and ultimately in determining the fetal weight at birth [10,11]. However, few studies examine how placental and fetal development are affected by rearing systems, especially regarding thermal stress, and how an environment with a pleasant microclimate can be beneficial.

The benefits of SP systems in regulating physiological systems in ewes during pregnancy compared with open pasture systems are not extensively discussed in the literature. We hypothesized that physiological and placental parameters are negatively affected when sheep are exposed directly to solar radiation. Thus, this study aimed to analyze the reproductive aspects and physiological changes of ewes kept in different rearing systems during pregnancy, to monitor the birth and the weight of their lambs, and to characterize the microclimatic variables of the systems in which the ewes were kept.

## 2. Materials and Methods

### 2.1. Study Area, Animals, and Experimental Design

The research was conducted at the Teaching, Research, and Extension of Sheep and Goat Farming Unit of the Federal University of Technology—Paraná, Campus Dois Vizinhos (UTFPR-DV). This project was approved by the Comissão de Ética no Uso de Animais (Animal Use Ethics Committee) (CEUA) of the UTFPR-DV, under protocol 2020-18, CEUA UTFPR.

Twenty-four Dorper × Santa Inês crossbred ewes, pregnant during a breeding season from 2 November to 16 December 2020 (with an average pregnancy duration of 50.4 ± 9.17 days), aged between 3 and 4.5 years, and with an average weight of 60 kg, were used in this study. They were divided into groups of 12 animals each and subjected to two treatments according to the rearing systems: the silvopastoral system (SP) and the open pasture system (OP). In the OP, the females remained in the open air without shade, and in the SP, the females had shade provided by trees at their disposal. In both treatments, the animals were kept in their respective paddocks during pregnancy; that is, rotation was not performed.

All the animals were kept on a pasture (*Panicum maximum Jacq* cv. Aruana) and supplemented with a concentrate of 1% of body weight (corn and soybeans); mineral salt and water were also offered ad libitum.

**Figure 1:**
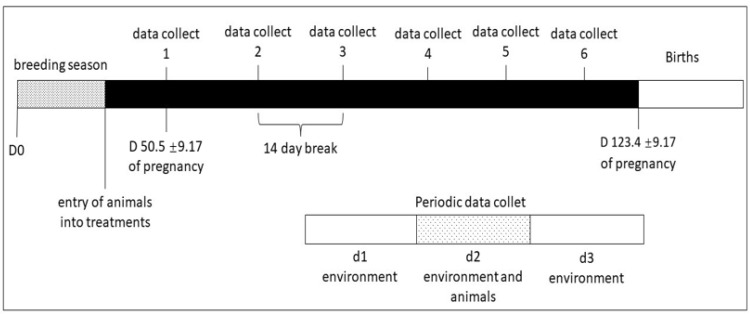
Representation of the breeding season scheme, periodic collections (main and microclimatic), and calving period.

Blood sample collections were carried out every 15 days from January to March 2021, with 6 collections in this period. The births took place in April and May (Figure 1).

It is important to note that, even in the breeding season, the people responsible for the blood sample collections were “introduced” to the sheep so that the animals could become accustomed to their presence. Therefore, no additional stress was created during the collections.

### 2.2. Microclimatic Variables

The microclimate of the two environments was evaluated by studying the environmental variables: air temperature (AT, °C), black globe temperature (BGT, °C), dew point temperature (DPT, °C), wind speed (WS, m s^−1^), and relative humidity (RH, %). These variables were collected for 12 h (7:00 am to 7:00 pm) on days when physiological variables and the animals’ blood were collected (Figure 1).

The temperature variables (AT, BGT, DPT) and RH were measured using Onset^®^ data loggers (HOBO U12-013), with a temperature measurement range between −20 and 70 °C, with an accuracy of ±0.35 °C and a relative humidity measurement range between 5 and 95%, with an accuracy of ±2.5%.

These devices were installed at 1.5 m in the SP (5 devices) and OP (1 device) areas. They had one internal channel and two external channels. The internal channel was used to couple a thermocouple cable (sensor) to the black globe (15 cm diameter hollow polyethene ball painted with matte black paint). WS was measured using a Mastech^®^ digital anemometer (MS6252A).

### 2.3. Thermal Comfort Indices

The radiant heat load (RHL) of the microclimatic variables of both the OP and the SP production systems was evaluated according to the equation proposed by Dixon [12]:

(1) $$RHL=\sigma {\left(MRT\right)}^{4}$$

where *RHL* was the radiant heat load (W m^−2^), σ was the constant of Stefan-Boltzmann (5.67 × 10^−8^, W m^−2^ K^−4^), and *MRT* was the mean radiant temperature (K), as described below:

(2) $$MRT=100\times {\left\{[2.51\times (WS^{\frac{1}{2}})\times \left(BGT-AT\right)]+[{\left(BGT\times 100^{-1}\right)}^{4}]\right\}}^{\frac{1}{4}}$$

where *WS* was the wind speed (m s^−1^), *BGT* was the black globe temperature (K), and *AT* was the air temperature (K).

### 2.4. Evaluation of Physiological Variables

The physiological variables analyzed were mean surface temperature, heart and respiratory rates, and rectal temperature. Measurements were taken from all the animals during the midday period. To collect these data, the animals were contained to minimize the stress caused by handling and not interfere with the data collection. The measurements were performed in the following order: heart and respiratory rates (HR and RR, respectively), surface temperature (ST), rectal temperature (RT), and finally, blood collection.

With the animals from the SP system, the collections were carried out in the paddock where they were kept. In the OP system, the animals were taken in pairs to a sheepfold and isolated in pens.

Two people were required to carry out the containment of the animals, one of whom was positioned on the right side of the animal while the other collected the data. HR and RR were measured by auscultation with a stethoscope. In HR, the stethoscope was positioned in the left thoracic region at the height of the aortic arch. The heartbeats were counted for 30 s, and then the value obtained was multiplied by 2 to obtain the number of beats per minute (beats. min^−1^). In RR, the stethoscope was positioned over the trachea to count the air passage activity for 30 s. The value obtained was multiplied by 2 to obtain the respiration rate per minute.

The average surface temperature of the animals was measured with a Flir TG165 infrared thermometer (with a measurement range of −25 to 380 °C, a precision of ±1.5% or 1.5 °C, and an emissivity of 0.90). Animals under the SP system were measured in the shade, and animals kept on the OP system were measured in direct sunlight. A person positioned 1 m from the animal measured the temperature at five points on the animal: head, neck, back, flank, and hind limb at thigh height. Then, the simple arithmetic average of the measured temperatures was calculated, as in previous studies [5,7].

Rectal temperature was measured using a mercury thermometer (5198.10, Incoterm, Brazil), which was introduced into the animal’s rectum so that the metallic tip remained in contact with the mucosa for one minute.

### 2.5. Peripheral Blood Sampling and Characterization of Leukocytes

Blood samples were collected between 11 a.m. and 1 p.m. by performing jugular vein punctures. They were stored in test tubes containing 3.2% sodium citrate (*m*/*v*), maintaining a ratio of 1:10 (citrate: blood) for later leukocyte characterization. The morphological analysis was carried out by adapting the methodology Thrall (2014) described. The smears were prepared from a drop of blood (approximately 20 μL) on a microscope slide, stained with panoptic dye (Newprov, Pinhais, Brazil). The morphological analysis of leukocytes was performed by optical microscopy, using a 100× objective lens together with immersion oil (Laborclin, Pinhais, Brazil). The quantitative analysis was carried out through the Neubauer Chamber using 4% Türk’s liquid (*m*/*v*) at a ratio of 1:20 (blood: Türk’s solution) in light microscopy (*n* = 15 per group).

### 2.6. Births and the Collection of Placentas

The ewes were kept in a suspended fold one week before the expected calving date. They were fed corn silage ad libitum, and the same supplementation conditions were applied (corn and soybean concentrate, mineral salt, and water). At delivery, the placentas were collected and taken to the laboratory. The lambs were weighed at birth and 10 days old. At the end of the pregnancies, 17 placentas were collected from the birth of 18 ewes. Pregnancies were classified as a single (1 lamb) or twins (2 lambs).

### 2.7. Placental Biometry

**Figure 2:**
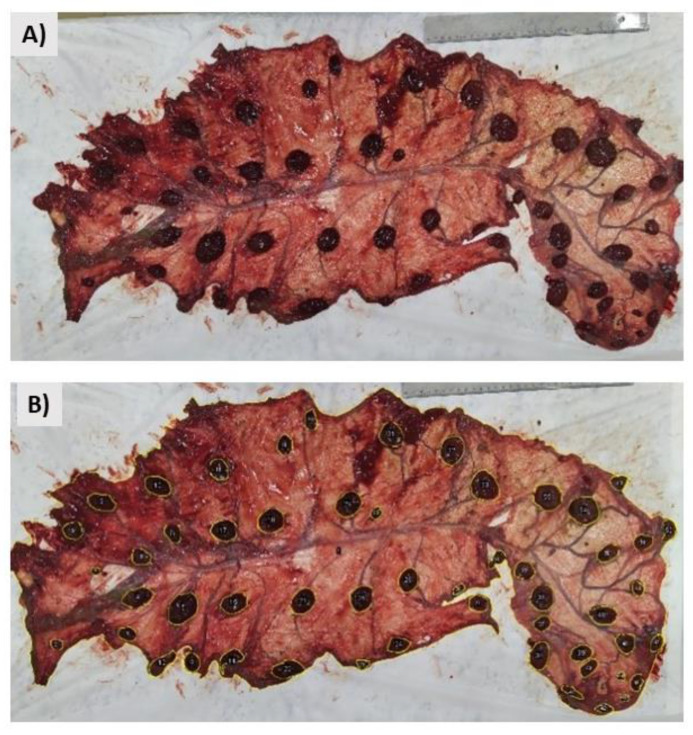
Representation of area measurement. (**A**) Placenta of single-gestation ewe. (**B**) Markings (yellow lines) and area and cotyledon numbering in ImageJ.

At the laboratory, the placentas were opened on a bench and photographed, and their cotyledons were counted. ImageJ software was used to define the area (membrane and cotyledons). The areas of interest were manually outlined using the “freehand” and “brush” tools, identified, and stored in the program. The data were processed, and the measurement was carried out, providing the areas delimited in cm². The rulers visible in the photographs (Figure 2) created a scale in centimetres on ImageJ.

### 2.8. Statistical Analysis

#### 2.8.1. Environmental and Physiological Variables

The experimental design was completely randomized, with 24 animals as biological replications (with 12 paddocks as experimental units and 2 animals per paddock as observational units). For the confirmatory analysis, mixed models were used, with time and treatment as fixed effects and date and paddock as random effects. The models were tested using the statistical software R [13] and the lme4 package [14]. The data were adjusted using ordinary least squares to enable an examination of the accuracy of the transformation of the response variables due to possible deviations from the assumptions of a linear model. In case of the need for transformations, the method of maximum likelihood was used. With the model adjusted, the data were analyzed using analysis of variance, and the type III F test was used for the model’s fixed effects. When significant, the Tukey test was used for multiple comparisons of means, with the level of significance declared when the *p*-value was greater than 0.05.

#### 2.8.2. Placental Biometry

To perform the statistical analysis, all the data were tested for normality and homogeneity (Shapiro-Wilk), considering a 2 × 6 factorial system (independent variable Production System × independent variable Time). The effects of Production System, Time, and System × Time interaction were verified. Normally distributed data were analyzed using Student’s t-test. Data that presented non-normal distribution were analyzed using the Mann-Whitney test. Dependent variables were considered: placental mass, placental area, and the number of cotyledons.

#### 2.8.3. Leukocyte Characterization

All the data were tested for normality and homogeneity of variances before being analyzed by two-way ANOVA, considering a 2 × 6 factorial system (independent variable Production System × independent variable Time). Effects of Production System, Time, and System × Time interaction were verified. The groups were compared using Tukey’s post-test with a significance level of 5%. Non-parametric data were transformed using the square root function and submitted to two-way ANOVA. Transformed data that did not meet the assumptions for analysis of variance were analyzed using the non-parametric Kruskal-Wallis’s test, followed by Dunn’s post-test using a significance level of 5%. Dependent variables were considered: total leukocytes, lymphocytes, neutrophils, monocytes, eosinophils, and basophils. All statistical analyses and graph productions were performed using Prism GraphPad version 7.0 software for macOS.

## 3. Results

### 3.1. Microclimate Characterization

Although variation was observed between the morning and afternoon shifts for all microclimatic variables, it was decided to focus on treatments since these were the primary goal of this experiment. The average AT for the SP was 0.9 °C below the average AT for the OP, as described in Table 1. As expected, a difference was observed in the AT from the shaded system compared to the OP (*p* = 0.0288), but no interaction was observed between these and the shifts. For RH, there was no variation between production systems and their interaction. As regards WS, no difference between treatments and their interactions with shifts was found. However, an effect on the GT was detected for the production systems (*p* = 0.0043) since the mean temperature of the OP was 2.2 °C above the temperature of the shaded pasture. For RHL, the only treatment effect observed was *p* = 0.0288 (Table 1), with an average variation of 34 W m^−2^ between the production systems. The lowest temperatures for both OP and SP were observed at 7 a.m. on 11 March 2021, while the higher temperatures for both systems were registered at 3 p.m. two days later (13 March 2021).

Finally, some microclimatic variables were correlated, proving AT’s dependence proportional to GT and RHL and inversely proportional to RH (*p* < 0.001). Also, through this correlation, it was noted that the WS had little influence on the other variables, having significant importance only in calculating the RHL (*p* < 0.001).

### 3.2. Thermoregulatory Variables

By observing the movement of the ewes’ flanks, the RR of ewes exposed to the sun was higher than that of ewes that remained in the shade (*p* < 0.001), since direct sun exposure increased the number of movements per minute by 38.5%. Furthermore, the HR of the sheep in the shaded system was considerably lower than the HR of the sheep in the open pasture (*p* < 0.001) since the blood-pumping rate is proportional to the heat dissipation rate (Table 2). The direct exposure of the animal to the sun significantly increased its MST by 2.8 °C compared to animals from the SP (*p* < 0.001). However, the RT was not affected by the presence of shade (*p* = 0.6742; Table 2). Additionally, regarding the RT, both systems had a normal distribution and were similar.

### 3.3. Leukocyte Characterization

**Figure 3:**
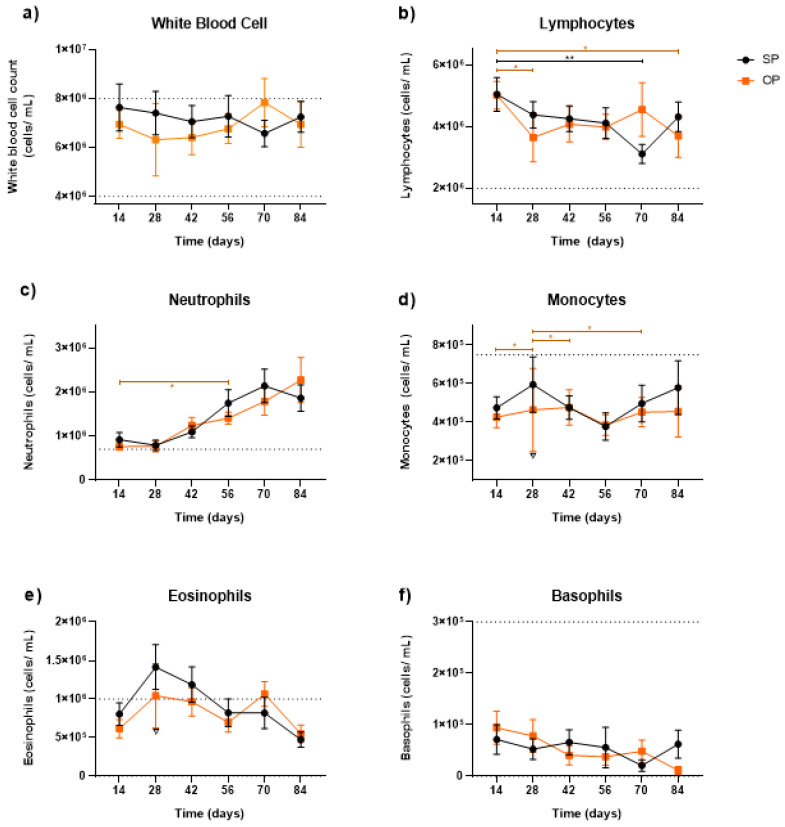
Mobilization of (**a**) total leukocytes; (**b**) lymphocytes; (**c**) neutrophils; (**d**) monocytes; (**e**) eosinophils; and (**f**) basophils (cells/mL of blood) during exposure to heat stress over the summer. * represents statistical variation over time (*p* < 0.005) of the OP system. ** represents statistical variation over time (*p* < 0.005) of the SP system. All values are represented as Mean ± SD.

The mobilization of white blood cells (WBCs) was not affected by the production system or by time and their interaction (Figure 3a). As regards lymphocytes, there was a difference in the cell count between days 14 and 70 for the SP (*p* = 0.0024) and between 14 and 24 and 84 for the OP (*p* = 0.0259; *p* = 0.0365, respectively). Even so, it was observed that there was a significant effect of time (*p* = 0.0055) and interaction (*p* = 0.0374) between the production systems (Figure 3b). The neutrophil cell count started near the lower reference limit and showed constant growth until day 84 for both systems. For the OP, however, a difference was spotted between days 14 and 56 (*p* = 0.0290). Finally, an effect of time (*p* < 0.0001) and interaction was observed (*p* = 0.0001; Figure 3c).

The OP system presented lower monocyte recruitment than the SP system on day 28 (*p* = 0.0119; Figure 3d). Nevertheless, this day contrasted significantly with days 14, 42, and 70 (*p* = 0.0476; *p* = 0.0387; *p* = 0.0206, respectively). As for the eosinophils, an effect of interaction between systems and time was observed (*p* = 0.0398; Figure 3e). The mobilization of the SP cells on days 28 and 42 were slightly above the upper reference limit, having a statistical distinction from the OP on day 28 (*p* = 0.0424). Finally, the only effect of interaction was detected for the basophils (*p* = 0.0325; Figure 3f).

### 3.4. Reproduction Variables

#### 3.4.1. Gestation and Lambs

The number of lambs born, the sex of the lambs, and their weight and weight gain ten days after birth were analyzed, but no system effect was observed (*p* = 0.6239; *p* = 0.9403; *p* = 0.5218; *p* = 0.3079, respectively). Although the median indicates a longer gestation for the SP system (145.5 days) as compared to the gestation for the OP system (140 days), there was no system effect on the duration of pregnancy and birth weight (*p* = 0.7954; *p* = 0.9441, respectively) (Table 3).

There was no effect of pregnancy on the duration of pregnancy and birth weight (*p* = 0.7715; *p* = 0.8519, respectively). When considering the weight and weight gain of the lambs ten days after birth, there was an effect of gestation (*p* = 0.0163; *p* = 0.0026, respectively) favoring simple pregnancy (Table 3). Interestingly, the production systems showed different types of placentation. While 22% of pregnancies in the OP group were twins and all were dichorionic, in the SP group 30% were twins, and all were monochorionic. There was no effect of the type of pregnancy in respect of the placental area, the placental mass, and the number of cotyledons (*p* = 0.2161; *p* = 0.3866; *p* = 0.2859, respectively). Numerically, monochorionic placentas had more cotyledons.

#### 3.4.2. Placental Biometry

There was no system effect (OP × SP) regarding the total area of cotyledons per placenta, the area, and the mass of the chorionic membrane (*p* = 0.8456; *p* = 0.7178; *p* = 0.9092, respectively). However, there was a significant correlation between the cotyledon area and the placental mass, but only for the OP group (*p* = 0.0148, R² = 0.8954). A positive correlation was also observed when the same analysis was performed, disregarding the rearing system (*p* = 0.0055, r² = 0.5934). However, there was no significant difference (*p* = 0.2541) between the OP and SP systems regarding the number of cotyledons, as shown in Table 4.

## 4. Discussion

### 4.1. Microclimate Characterization

Because of their wool, sheep suffer more from the effects of HS. Owing to the physicochemical properties of wool, sheep in high-temperature environments have a lower rate of loss of sensible heat by conduction and convection (body surface—environment), which makes it difficult for heat to be dissipated and for water to evaporate [15].

The SP system, characterized by a combination of trees, pasture, and animals in the same space, aims to improve the thermal comfort of these animals through the shade provided by trees [16]. The microclimate generated has a positive influence on the grazing time [17] and the rumination of sheep [7], reduces the air temperature and increases the air humidity [18], and reduces the RHL thanks to the radiative interception provided by the canopy of trees [19,20]. The OP, which is the most common rearing system in the subtropical areas, does not present any trees (i.e., there is no shade), and this leads to higher air and grass temperatures alongside high RHL values.

Both systems remained within the parameters of thermal comfort for sheep, with temperatures ranging between 20 and 30 °C [21] and relative humidity close to 60% [22]. However, our relative humidity values were slightly higher than the reference, while the air temperature was lower than in our previous study [7]. Recent studies [23,24] also showed that the OP had higher AT and RH values when compared to the SP, which corroborates our findings. As regards WS, although our results did not show any difference, other studies found that WS in the OP was higher than in the SP [25].

According to Silva [26], RHL values above 570 W m^−2^ indicate a harsh environment for female animals. As regards high air temperature and humidity, higher RHL values were expected in the OP than in the SP; this was confirmed by the variance of 34 W m^−2^ between both systems. From this, the microclimate of both production systems was stressful for the animals, despite the OP having significantly higher values than the SP. Therefore, this exposure may cause discomfort to animals in direct contact with the pasture. Recent studies [7,20,27] analyzed the behaviour of adult females and their lambs and related direct sun exposure with increased GT and CTR; this affects them negatively and corroborates the premise that the OP provides a more stressful environment for the animals that are kept there. Our findings regarding RHL also concur with those previously described in the literature [19].

### 4.2. Physiological Variables

The physiological variables of sheep that can be used to assess whether they are under heat stress are respiratory rate, heart rate, and rectal temperature [28]. Thus, the ewes will trigger physiological mechanisms to improve homeostasis as the ambient temperature rises. In the present study, sheep from the OP system have a higher HR and RR, which is an apparent sign of heat stress. Similar results were found by Nejad and Sung [29], who, when analyzing the physiological parameters of sheep subjected to heat stress and limited water supply, observed that sheep without heat stress had lower RR values and gasping scores (*p*-value 0.02 and 0.03, respectively).

Despite the differences between the other physiological variables, TR is the main variable used to identify the state of the thermal stress of the animal. Therefore, even though the sheep were in challenging microclimatic conditions, they could maintain their body temperature within the normal range of this variable, which may be linked to the increase in HR and RR. The RT of both systems was similar, in contrast to that presented in the literature, where ewes under heat stress present a high RT [30,31,32].

The surface temperature can be used as a parameter for evaluating the thermal comfort of sheep, as there is a correlation between environmental indices and rectal and skin surface temperature [33]. Thus, the highest value of MST was detected for the OP system, where the highest temperatures were observed.

### 4.3. Leukocytes

We also hypothesized that heat stress (HS) negatively affects the sheep’s immune system. However, our results showed that the recruitment of WBCs was not affected by direct exposure to solar radiation (Table 1). Although some studies analyze the physiological [5], behavioural [7], and biochemical [34] response to heat stress in a silvopastoral system, the literature lacks information regarding the mobilization of leukocytes in adult sheep, whether heat-stressed or in a shaded production system. HS is commonly known to affect leukocyte concentration in broiler chickens [35], dairy cattle [36], human workers [37], and even Australian abalone [38].

Our results in respect of WBC are in accordance with what is described in the literature for heat-stressed and non-heat-stressed sheep. However,, we observed higher eosinophil numbers along with higher monocyte concentration for a time. Karthik [32] observed higher WBC populations in sheep in an extensive heat-stressed system compared to an intensive one. Yet, the harsh environment did not affect the subpopulations of WBCs (neutrophils, lymphocytes, eosinophils, monocytes, and basophils). Contrary to our results, Wojtas et al. [34] discovered that total leukocyte mobilization in sheep was influenced by the movement and temperature of the air, being in lower concentrations when under high temperatures. Caroprese et al. [39] presented an alternative to combat the negative effects of HS in sheep based on supplementation with polyunsaturated fatty acids. Their results showed that supplementing flaxseed may improve the cell-mediated immune response in sheep exposed to solar radiation. Swanson et al. [40] found that heat-stressed lambs presented higher WBC, monocyte, and granulocyte concentrations in blood plasma compared to non-heat-stressed lambs. At the same time, Liu et al. [41] showed that lymphocyte count was reduced in the stressed environment. Cortisol is a hormone produced by the pituitary gland in response to HS exposure. It is one of the most important factors leading to reduced leukocyte mobilization [42]. It is possible that in the present study, despite the low but significant difference between the systems’ RHLs, the increase in the cortisol hormone was not enough to significantly affect the WBC concentration in the plasma. However, more studies involving plasma cortisol analysis are needed to confirm this hypothesis.

### 4.4. Gestation and Lambs

In several studies, the effects of heat stress on the body weight at birth of ewes and other ruminants are observed [32,43,44,45]. When comparing the birth weight of lambs in a controlled environment with the birth weight of lambs from ewes exposed to heat stress, a lower weight is noted in the second group; however, this article does not present this system effectively. According to Marai et al. [43], this inferiority in weight is due to a disturbance in placental growth generated by heat, which affects fetal development and, consequently, the birth weight of the lambs.

There was no system effect on the number of lambs born. In contrast, Van Wettere et al. [45] found fewer lambs born to mated ewes exposed to heat stress in their work. Their studies show harmful alterations in the fertility and fecundity of ewes and the production and quality of semen in the rams. Other studies indicate that non-heat-stressed ewes are 2.43 times more likely to become pregnant than heat-stressed ewes [44].

The average weight gain in goats eight days after birth is lower than in sheep raised in heat-stress systems; this may be associated with indications of low placental efficiency [46]. However, the present study does not show any statistically significant system effect on weight and weight gain ten days after birth. In cows, Casamassima et al. [47] find that heat stress significantly reduces milk production and quality, which negatively impacts lamb growth.

Heat stress can reduce the duration of pregnancy in cows and goats, directly impacting the reduction in birth weight [48,49]. However, the present study does not show a system effect on the duration of the gestation of ewes, even with a lower mean of the OP system (140 days) compared to the SP system (145.5 days).

Interestingly, the production systems showed different types of twin placentation. While the pregnancies in the OP group were all dichorionic, those in the SP group were all monochorionic. Although studies such as those conducted by Dwyer et al. [50] and Özyürek and Türkyilmaz [51] state that twin gestation lambs have an increase in placental weight and the number of cotyledons, the type of pregnancy showed no effect on the area, the mass of the placenta, and the number of cotyledons.

In humans and ewes, monochorionic twin pregnancies have complications arising from vascular anastomoses, which is not observed in dichorionic pregnancies. Additionally, in twin pregnancies, there is unequal sharing of the placenta by the fetuses [52,53]. In ewes, this can lead to the birth of unequal-sized lambs, which impacts their survival and development.

### 4.5. Placental Biometry

There was no system effect (OP × SP) for the total area of cotyledons per placenta and the area of the chorionic membrane (*p* = 0.8456; *p* = 0.7178, respectively). However, there was a significant correlation between the cotyledon area and the placental mass, but only for the SP group (*p* = 0.0148, r² = 0.8954). When the same analysis was performed disregarding the rearing system, a positive correlation was also observed (*p* = 0.0055, r² = 0.5934), allowing the inference that the cotyledon area and the placental mass are correlated.

Placental efficiency can be defined as the placenta’s capacity to support the fetus’s growth: the greater the weight of the fetus in relation to the placental weight, the greater the placental efficiency [54]. Since placental vascular architecture is closely related to placental efficiency, it is essential to evaluate placental biometry to obtain correlations.

Decreased placental development also limits the supply of oxygen and nutrients, and this leads to decreased fetal plasma glucose and fructose rates and reduced fetal growth rates [55].

Such results corroborate what is described in the literature, since there is proportionality between the placental mass and the number of cotyledons [50]. A greater number of cotyledons can be related to a greater passage of nutrients between the fetus and the mother [56]). However, there was no significant difference (*p* = 0.2541) between the OP and SP systems regarding the number of cotyledons (Figure 3).

The increase in placental weight in multiparous mothers may be due to the expansion and vascularization of the uterus after multiple pregnancies and advancing maternal age [50]. The age of the ewe and its reproductive maturity can significantly influence placentation [57]. Finally, drawing on our findings, we recommend using the silvopastoral system to raise ewes and lambs to minimize the effects of heat stress on these animals.

## 5. Conclusions

No significant differences between rearing systems (OP and SP) regarding gestation variables and lambing were observed. We observed some interesting points concerning the types of gestation (twin pregnancy and simple pregnancy); each system presented just one example of twin pregnancy; however, this did not affect the remaining variables.

## Figures and Tables

**Table 1:** Microclimatic variables between rearing systems throughout the summer (mean ± SD; median, maximum, minimum).

Variables		Open Pasture (OP)	Silvopastoral System (SP)	*p* Value
Air temperature (AT, °C)	Mean ± SD	26.9 ± 0.41 A	26.0 ± 0.38 B	0.0288
	Minimum	17.0	17.4	
	Median	26.2	26.2	
	Maximum	34.3	33.3	
Relative humidity (RH, %)	Mean ± SD	67.2 ± 3.42 A	68.4 ± 3.37 A	
	Minimum	28.1	28.3	0.4011
	Median	69.5	69.5	
	Maximum	93.7	93.9	
Wind speed (WS, m s^−1^)	Mean ± SD	1.25 ± 0.20 A	1.02 ± 0.13 A	0.0939
	Minimum	0	0	
	Median	0.86	0.86	
	Maximum	4.04	5.69	
Grass temperature (GT, °C)	Mean ± SD	25.6 ± 0.44 A	23.4 ± 0.37 B	0.0043
	Minimum	11.0	9.6	
	Median	23.8	23.8	
	Maximum	37.6	31.4	
Radiant heat load (RHL, W m^−2^)	Mean ± SD	610 ± 12.3 A	576 ± 10.9 B	0.288
	Minimum			
	Median			
	Maximum			

Means with different letters within each factor differ among themselves (Tukey’s test; *p* < 0.05). AT (air temperature); RH (relative humidity); WS (wind speed); GT (grass temperature); and RHL (radiant heat load).

**Table 2:** Physiological variables between rearing systems throughout the summer (mean ± SD; median, maximum, minimum).

Variables		Open Pasture (OP)	Silvopastoral System (SP)	*p* Value
Heart rate (HR, beats min.^−1^)	Mean ± SD	116 ± 4.85 A	100 ± 4.84 B	<0.001
	Minimum	64	48	
	Median	117	97	
	Maximum	178	166	
Respiratory rate (RR, mov. min.^−1)^	Mean ± SD	104.1 ± 10.0 A	76.4 ± 10.1 B	
	Minimum	24	36	<0.001
	Median	102	68	
	Maximum	198	182	
Rectal temperature (RT, °C)	Mean ± SD	39.35 ± 0.06 A	39.33 ± 0.06 A	0.06742
	Minimum	38.4	38.4	
	Median	39.4	39.3	
	Maximum	40.0	40.4	
Mean surface temperature (MST, °C)	Mean ± SD	32.8 ± 0.76 A	30.0 ± 0.76 B	<0.001
	Minimum	26.0	25.3	
	Median	33.4	29.7	
	Maximum	44.4	38.5	

Means with different letters, within each factor, differ among themselves (Tukey’s test; *p* < 0.05). HR (heart rate); RR (respiratory rate); RT (rectal temperature); and MST (mean surface temperature).

**Table 3:** Mean values (mean ± SEM) of the reproductive variables and lamb’s weight between rearing systems.

Variables	Open Pasture (OP)	Silvopastoral System (SP)	*p* Value
Duration of pregnancy (days)	138.6 ± 12.79	137.6 ± 16.39	0.7954
Birth weight (lambs) (kg)	3.937 ± 0.4506	3.921 ± 0.5943	0.9441
Ten days weight (lambs) (kg)	5.788 ± 0.8883	6.124 ± 1.1257	0.5218
Weight gain (lambs) (kg)	1.774 ± 1.010	2.186 ± 0.7481	0.3079

Tukey’s test; *p* < 0.05.

**Table 4:** Mean values (mean ± SEM) of the placental biometry of ewes kept in the silvopastoral system (SP) and open pasture (OP).

Variables	Open Pasture (OP)	Silvopastoral System (SP)	*p* Value
Membrane area (simple pregnancy) (cm^2^)	2296 ± 473.6	2298 ± 490.4	0.9578
Cotyledon area (simple pregnancy) (cm^2^)	234.7 ± 69.59	226.4 ± 68.62	0.8456
Number of cotyledons	60.22 ± 22.06	70.75 ± 12.61	0.2541
Placental mass (kg)	0.3423 ± 0.08057	0.3370 ± 0.1084	0.9092

(Mann-Whitney test).

## Data Availability

Not applicable.
